# Assessment of Mandibular Surface Area Changes in Bruxers *Versus* Controls on Panoramic Radiographic Images: A Case Control Study

**DOI:** 10.2174/1745017901814010753

**Published:** 2018-09-28

**Authors:** Lakshmi Padmaja Satheeswarakumar, Tatu Joy Elenjickal, Shashi Kiran Mohan Ram, Kartheesan Thangasamy

**Affiliations:** Department of Oral Medicine and Radiology, Sree Mookambika Institute of Dental Sciences, Kulasekharam, Kanya Kumari District, Tamil Nadu, India.

**Keywords:** Bruxism, Condyle, Sleep bruxism, Mandibular surface area, Temporomandibular joint disorders, Panoramic radiographic images

## Abstract

**Background:**

Bruxism is the commonest of the many parafunctional activities of the masticatory system. Opinions on the causes of bruxism were numerous and widely varying. It can occur during sleep as well as wakefulness. Bruxism was for long considered a major cause of tooth wear. Other effects of bruxism may include tooth movement and tooth mobility, as well as changes in oral soft tissues and jaw bone. Since the exact etiology and manifestations are unclear, it was difficult to diagnose Bruxism. In this study, we evaluated the area change as measured from digital panoramic radiographs that can occur in the lower jaw bone in those with Bruxism and compared the results with non-bruxers.

**Aims and Objective:**

To determine the surface area changes of the mandible, condylar and coronoid processes in Bruxers from Panoramic radiographs and to compare and contrast the changes with age and gender matched controls.

**Materials and Methods:**

The study was conducted in the department of Oral Medicine and Radiology. The total sample size was 40. The sample was divided into two groups, Bruxers and non-bruxers with 20 subjects in each group. Healthy volunteers aged between 20- 30 years diagnosed with Bruxism and Healthy volunteers aged between 20- 30 years diagnosed without Bruxism were included in group II (Non-Bruxers). Bruxchecker was made use of in confirming the Bruxism in Group I. The Panoramic radiograph was used as the imaging modality for the study. The measurements were made with the help of software, Image J. All the measurements were tabulated and statistical analysis was made using ANOVA (Post hoc) followed by Dunnett’s test and unpaired t test.

**Results:**

A comparison of the mandibular surface area as a whole and also condylar and coronoid processes individually were carried out. Significant results were obtained in case of condylar and coronoid processes between the two groups. The surface area of condylar process of Group I was found to be lower than that of Group II. The surface area of the right coronoid process of group I was found to be less when compared to that of group II but the values of the left coronoid process of group I was found to be more when compared with group II. The surface area of the mandible showed no significant difference between the groups. There was significant difference between the genders in case of mandible, condyle and coronoid. The surface area of mandible and condylar process was found to be lower in female when compared to male. The surface area of coronoid process was found to be more in case of females when compared to that of males in Group I.

The results of our study show that while the overall surface area of bruxers remain unaffected when compared to controls, the condylar and coronoid process show significant change. The hitherto belief that the primary brunt of bruxism is borne by the masseter would require a revisit since alteration in tonicity of the masseter would reflect in surface area change of the mandible as a whole. An increase in the surface area of the coronoid process in bruxers was observed in our study which could be attributed to altered activity of the temporalis, a muscle largely responsible for the posture of the mandible. This could imply that bruxers show alteration in temporalis activity which would explain several clinical manifestations such as headache, neck pain, shoulder pain and altered posture and so on which we have observed in the clinical practice of neuromuscular dentistry. Further studies examining the activity of the temporalis and masseter would further corroborate our findings and form the basis for future research in this arena.

**Conclusion:**

This original research was carried out to assess the surface area changes in mandible and condylar and coronoid processes of Bruxers and non-bruxers. The results showed significant changes in the surface area of condylar and coronoid process in Bruxers when compared to the controls. This is an area of study with paucity of available literature. This study would be a stepping stone for future studies in this arena.

## 1 INTRODUCTION

Bruxism is the movement disorder with grinding and clenching of dentition as characteristic features. Bruxism is a detrimental parafunctional activity resulting in excessive stress on the stomagnonathic system. The word Bruxism is of Greek origin, derived from the word “brygmos”, meaning “gnashing of teeth” [ 1]. The cause for Bruxism can be broadly divided into three: Psychosocial, pathophysiological and peripheral factors [2]. Bruxism can occur while the individual is awake or during sleep. These are referred to as ‘Awake Bruxism’ and ‘Sleep Bruxism’ respectively. Sleep Bruxism has been defined as a stereo type movement disorder occurring during sleep and characterised by tooth grinding and clenching [ 3 ]. Sleep Bruxism has a prevalence rate of 8-16% in the adult population. Bruxism is believed to have a multifactorial etiology. Among the pathophysiological factors, the ‘arousal response’ is believed to be in pivotal. Arousal response is a change in the depth of sleep during which the individual transients from deep sleep to a stage of lighter sleep or wakefulness. This change is associated with increased heartrate, altered respiratory rate and increased muscle activity and Bruxism is believed to be a part of this response [ 4 ]. Psychosocial factors have long been associated with Bruxism with bruxers showing depression, increased levels of hostility and stress sensitivity [ 5 ] . Among the peripheral factors harmonious occlusion during centric relation and occlusion is imperative [ 6 ]. 

Alterations in the bone architecture among individuals with parafunctional habits is not an extensively studied area. Bruxism by its very nature exerts tremendous continued force on the odontogenic apparatus and its supporting osseous structures. Long term pathological forces of this nature is found to have an effect on these structures and this forms the core concept of our research. Several means have been utilised to assess Bruxism including quetionnaires, electromyographic studies of masticatory muscles as well as intraoral appliances such as bruxcheckers. In our study we have chosen to use bruxcheckers to identify bruxers and panoramic radiographs to assess osseous changes in bruxers. This was done keeping in mind the widespread availability of panoramic imaging facilities and cost effectiveness and ease of availability of bruxcheckers.

## 2 MATERIALS AND METHODS

The study was approved by institutional ethical committee. The study was conducted in the department of Oral Medicine and Radiology, Sree Mookambika Institute of Dental Sciences, Kulasekharam, Kanyakumari district to assess the mandibular surface area changes in Bruxers versus controls on Panoramic radiographic images. The total sample size was 40.

The sample was divided into two groups, Bruxers and non-bruxers with 20 subjects in each group. Healthy volunteers aged between 20- 30 years diagnosed with Bruxism, Dentate individuals, with minimum of 28 teeth except the third molars. Patients with symmetrically intercuspating teeth were included in group I (Bruxers) and Healthy volunteers aged between 20- 30 years diagnosed without Bruxism, Developmental anomalies and Syndromes affecting the size and shape of the mandible, Bone altering diseases such as osteo-dystrophies and also including pathologies of the mandible such as neoplasms, cysts and fractures, Pregnant patients, Patients with asymmetrically intercuspating teeth, with periodontal disease, undergoing / undergone orthodontic treatment, Age below 20 years and above 30 years with para functional habits were included in group II (Non Bruxers).

**Fig. (1):**
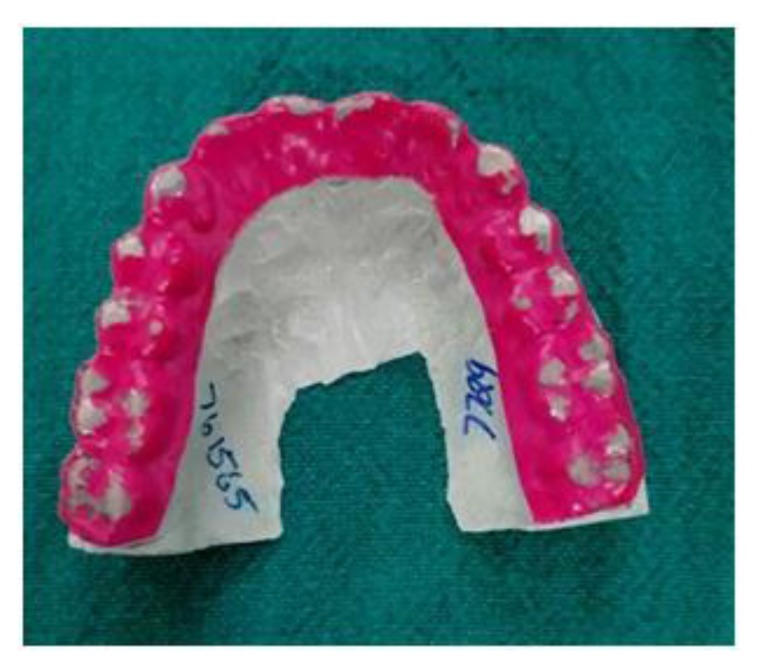
Bruxchecker after the use,

**Fig. (2):**
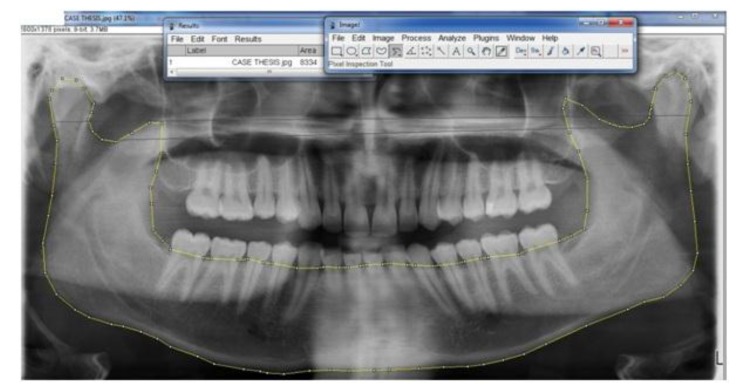
Mandible tracing using Image J.

**Fig. (3):**
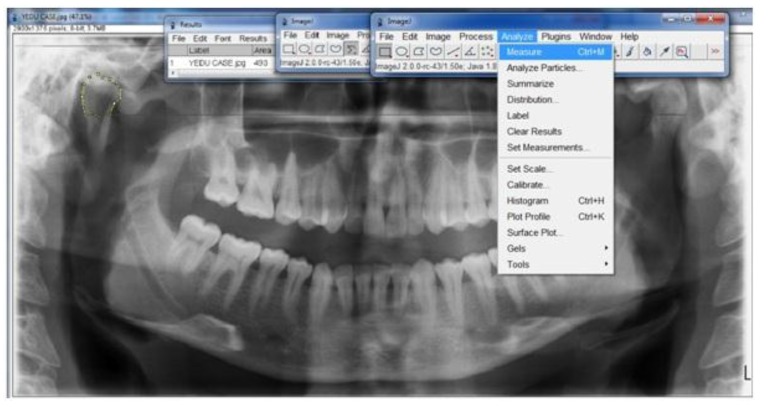
Condylar tracing using Image J.

**Fig. (4):**
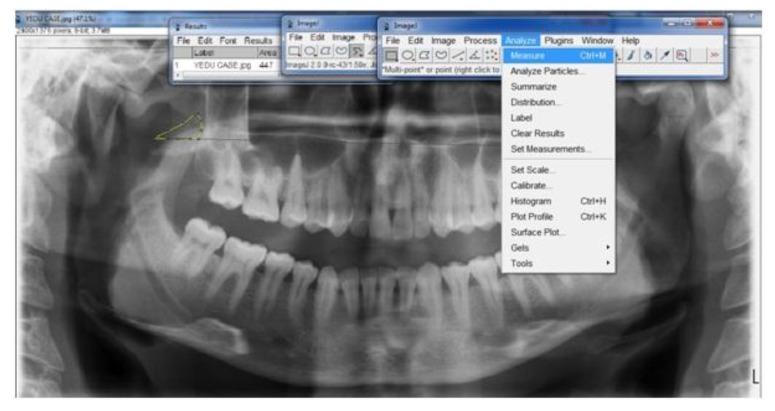
Coronoid process tracing using Image J.

Patients with a clinical history suggestive of Bruxism were taken and a 0.1 mm bruxchecker were customised using a biostar machine. Bruxchecker is heated to 230°C using a vacuum deep-drawing device (Biostar, Scheu Dental) and deep drawn for 15 s over a suitable dental plaster model. The suspected case was asked to wear the bruxchecker during sleep and included or excluded as Bruxer based on the wearing on the bruxchecker (Fig. **1**). Patients identified as bruxers were subjected to an OPG. The OPGs were taken as a part of annual dental examination. The images were acquired using Planmeca Proline XC Digital Orthopantomograph Machine, Finland. The exposure parameters were selected according to the patients variability in size and it ranges from 68 kv to 80 kv and 5 Ma to 8mA with a magnification factor of 15%. The external surface area of the mandible was measured using “Image J” software. The mandible was traced along the outermost margins of the mandibular image, and then through the buccal aspect of the alveolar crest (Fig. **2**). A straight line was drawn across the pterygoid fovea on both sides and the condyle is traced along its outermost margins that extends till the straight line (Fig. **3**). A straight line was drawn connecting the deepest point of sigmoid notch on both sides and the coronoid is traced along its outermost margins that extends till the straight line (Fig. **4**) The tracings were counterchecked by two experts in the field (Radiologists). The area thus obtained from the “Image J” software. The data of both case and control were entered in to the data sheet. The surface area of the mandible, condyle and coronoid of Bruxers versus controls were compared. The results were obtained by the statistical analysis ANOVA (Post hoc) followed by the Dunnett’s test and unpaired *t*-test.

## 3 RESULTS

The present study was conducted to assess the mandibular surface area changes in bruxers and nonbruxers. It was carried out on a study group comprising 20 healthy individuals as controls in comparison with 20 bruxers (10 males and 10 females). A comparison of the mandibular surface area as a whole and also condylar and coronoid processes individually were carried out. The mean surface area of two groups are found to be 7315.30 ± 3.67 and 7347.03, which showed no significant difference (Table **1**). The mean condylar surface area of two groups are found to be 425.15 ± 5.83 and 427.85 ± 5.58 for right and left condylar processes of bruxers respectively and 455.80 ± 4.36 and 467.45 ± 5.71 for the right and left condylar processes of nonbruxers respectively. The *P* value was found to be 0.04 that revealed significant reduction in the surface area of the condylar process in Group I when compared to Group II (Table **2**). The mean coronoid process surface area of two groups are found to be 497.65 ± 6.87 and 525.65 ± 7.23 for right and left coronoid processes of bruxers respectively and 502.50 ± 3.96 and 516.70 ± 4.14 for the right and left coronoid processes of nonbruxers. The *P* value was found to be 0.04 that revealed significant change in the surface area of the coronoid process. The surface area of the right coronoid process of group I was found to be less when compared to that of group II but the values of the left coronoid process of group I was found to be more when compared with group II (Table **3**). 

A Comparison of the mean mandible surface area between the genders within the groups was performed. The mandible surface area of female were found to be lower when compared to that of men in both the groups (Table **4**). A Comparison of mean condylar process surface area between the genders within the groups was performed. The condylar surface area of female were found to be significantly reduced when compared to that of male in both the groups (Table **5**). A Comparison of mean coronoid process surface area between the genders within the groups was carried out. The surface area of left coronoid process of Group I male and right coronoid process of Group II male were found to be lower when compared to that of female (Table **6**).

A Comparison of mean mandible surface area between the genders between the groups were performed. On comparison of the mean values there were no significant difference observed between the genders (Table **7**). A Comparison of mean condylar process surface area between the genders between the groups. The condylar surface area of Group I females and males were found to be significantly reduced when compared with the Group II females and males (Table **8**). A Comparison of mean coronoid process surface area between the genders between the groups. The surface area of coronoid process of Group I female left side was found to be higher than that compared to the Group II females. The surface area of coronoid process of Group I male right side was found to be higher than that compared to the Group II males (Table **9**). 

## 3.1 Statistial Analysis

The data was expressed in number, percentage, mean and standard deviation. Statistical Package for Social Sciences (*SPSS* 16.0 version) used for analysis. ANOVA (Post hoc) followed by Dunnett’s test and unpaired *t*-test applied to find the statistical significant between the groups.

## 4 DISCUSSION

In this study we have tried to evaluate the changes in the mandible, in those with Bruxism and compared the results with that of non bruxers. The subjects were grouped according to the presence or absence of Bruxism based on the pattern recorded on the Bruxchecker^TM^. Once the subject was confirmed to be a Bruxer, they were subjected to panoramic imaging. The surface area changes of the mandible as a whole, coronoid and condylar process separately were evaluated in both the groups. The Image J software was utilised for the tracings and the measurements.

Image J is a public domain image processing software that was adapted in many scientific studies for image processing. The software was made use of in various medical as well as dental researchers for image analysis. Lemos AD *et al*., and Girish V *et al*., and various other researchers carried out their studies using this analytic software [ 7, 8 ] . 

From the outcome of this study, the surface area of the condylar process in bruxers was found to be reduced. The changes in the condylar process and its association with that of parafunctional habits were studied and proved in various publications. Yamada K *et al*., in a study have evaluated the association between condylar bony changes and parafunctional habits which concludes that greater the number of parafunctional habit, the higher the risk of developing condylar bony change and deterioration of the temporomandibular joint [ 9 ] . 

In a study by Nagahara K *et al*., in 1999, they have analyzed the biomechanical reactions in the mandible and temporomandibular joint during clenching under various restraint conditions. A three-dimensional finite element model of the mandible, including the TMJ, was created for test purposes. The result of the study showed that, under any restraint conditions, displacement was greatest on the surface of the condyle and less on the articular disc. This substantiates our study results as we saw conspicuous changes in the condylar process [ 10 ] . The study conducted by Dias GM evaluated the presence of degenerative bone changes of the temporomandibular joint in individuals suffering from Sleep Bruxism and found positive correlation and also proved that degenerative bone disorders was high among women [ 11 ].

The mandibular surface area as a whole did not show any significant difference between bruxers and non-bruxers. However, the surface area of the female mandible was found to be less when compared to that of male mandible. Our study shows no significant change in the surface area of mandible of bruxers and controls but there was significant change in the surface area of the mandible when the genders were compared. The surface area of male was found to be more when compared to female. This is in expected lines with most of the anthropometric studies and corroborates with the results of earlier studies in this field like those by Liu YP *et al*., [ 12 ].

The coronoid process was found to have a marginal increase in its surface area in bruxers when compared to that of non bruxers. The hyperactivity of the masticatory muscle especially the temporalis could be the logical explanation of this hyperplasia. A study by Kim SM *et al*., postulates a hypothesis that temporalis hyperactivity leads to coronoid hyperplasia [ 13 ] . 

From clinical experience, the general consensus is that there would be severe changes in the masseter muscle in bruxism. There are a multitude of studies that confirm the role of masseter hypertrophy in the increase in size of mandible mainly at the gonial angle [ 14 ].

In our study, there was no obvious change in the surface area of the mandible in bruxers when compared to that of controls. This finding suggests that the masseter activity is of no/less significance in bruxers. But there was increase in the surface area of coronoid process that squarely puts the temporalis muscle in the dock, temporalis being the muscle for posturing of the mandible. Our contention is that bruxism led to the hyperactivity of the temporalis muscle and in turn bone deposition at the coronoid process leading to the perceived change. Clinical data suggests that bruxers manifest with headache and neck pain rather than facial pain. These suggest a positive correlation of the finding that it is the temporalis that is involved and affected more in bruxism than the masseter muscle. This aspect will need to be studied further with the aid of surface Electromyography.

There are differences in opinions and findings regarding the masticatory muscle activity in Bruxers. The result of a study by Palinkas *et al*., revealed that Sleep Bruxism (SB) negatively altered the masticatory muscles functions. Based on the results, the authors concluded that individuals with Sleep Bruxism (SB) showed decreased Electromyographic activity in the masticatory muscles [ 15 ].

There are various studies that are in consensus with our study. A study conducted by Hoyos JAA *et al*., on the effect of Sleep Bruxism on masseter and temporalis before and after selective grinding showed remarkable reduction in the muscle activity after selective grinding. The surface electromyography of temporal muscle showed a statistically significant reduction of action potentials after the selective grinding [ 16 ]. 

Since our study churned out a few unanticipated results, this can be considered as a forerunner for future studies in this field. Advanced techniques for assessing muscle activity and volumetric analysis can provide more insight into bruxism and its alliterations on the neuromuscular and musculoskeletal system.

## CONCLUSION

This was an original research that was carried out to assess the surface area changes in mandible and the condylar and coronoid process of Bruxers and controls. The results showed significant changes in the surface area of condylar and coronoid processes in Bruxers when compared to the controls. The change in the condylar and coronoid processes in Bruxism is an open area of research. This study is an initial attempt to assess the bony changes in Bruxers which is seldom carried out by other researchers. There is literature that proves the relationship between TMD and bruxism but the changes in the individual bony component has not been extensively studied. Also in this study, the importance of the Temporalis muscle in Bruxism was revealed that paves way for future research in this arena.

This study would be a stepping stone for the future studies in this field. We made use of the simple techniques for assessing bruxism as well as simple imaging modality and also easy and simple image analysing tool. Advanced techniques and modes can be used in future to revaluate the results and to establish new outcomes.

## Figures and Tables

**Table 1:** Comparison of mean mandible surface area of different groups.

**Groups**	**Mandible Surface Area (mm) (MEAN±SD)**	***P* value **
**Group-I**	7315.30±3.67	**0.67**
**Group-II**	7347.03±2.99	

(*p*>0.05 no significant difference compared Group-I with Group-II)

**Table 2:** Comparison of mean condylar process surface area of different groups.

**Groups**	**Condylar Process (mm) (MEAN±SD)**			
	**Right**	***P* value**	**Left **	***P* value**
**Group-I**	425.15±5.83	**0.04**	427.85±5.58	**0.04**
**Group-II**	455.80±4.36*		467.45±5.71*	

(*p<0.05 significant compared Group-I with Group-II).

**Table 3:** Comparison of mean Coronoid process surface area of different groups.

**Groups**	**Coronoid Process (mm) (MEAN±SD)**			
	**Right**	***P* value**	**Left **	***P* value**
**Group-I**	497.65±6.87	**0.04**	525.65±7.23	**0.04**
**Group-II**	502.50±3.96*		516.70±4.14*	

(*p<0.05 significant compared Group-I with Group-II).

**Table 4:** Comparison of mean mandible surface area between the genders within the groups.

**Gender**	**Group-I Mandible Surface Area (mm) (MEAN±SD)**	***P* value**	**Group-II Mandible Surface Area (mm) (MEAN±SD)**	***P* value**
**Female **	7195.30±2.11	**0.04**	7176.50±2.90	**0.03**
**Male**	7435.20±2.54*		7517.40±2.01*	

(*p<0.05 significant compared Female with Male).

**Table 5:** Comparison of mean condylar process surface area between the genders within the groups.

**Gender**	**Group-I Condaylar process surface area (mm) (MEAN±SD)**		**Group-II Condaylar process surface area (mm) (MEAN±SD)**	
	**Right**	**Left**	**Right**	**Left**
**Female **	391.30±2.45	398.40±2.08	454.00±3.46	439.50±4.36
**Male**	459.00±6.36*	457.30±6.49*	457.60±5.29	495.40±5.69*

*p<0.05 significant compared Female with Male.

**Table 6:** Comparison of mean coronoid process surface area between the genders within the groups.

**Gender**	**Group-I Coronoid Process Surface Area (mm) (MEAN±SD)**		**Group-II Coronoid Process Surface Area (mm) (MEAN±SD)**	
	**Right**	**Left**	**Right**	**Left**
**Female **	495.10±5.19	537.80±6.18	512.70±1.51	516.20±3.65
**Male**	500.20±8.51	513.50±8.29*	492.30±5.34*	517.20±4.79

(*p<0.05 significant compared Female with Male).

**Table 7:** Comparison of mean mandible surface area between the genders between the groups.

**Gender**	**Group-I Mandible Surface Area (mm) (MEAN±SD)**	**Group-II Mandible Surface Area (mm) (MEAN±SD)**	***P* value**
**Female **	7195.30±2.11	7176.50±2.90	**0.56**
**Male**	7435.20±2.54	7517.40±2.01	**0.78**

(*p*>0.05 no significant difference compared Group-I with Group-II).

**Table 8:** Comparison of mean condylar process surface area between the genders between the groups.

**Gender**	**Group-I Condaylar Process Surface Area (mm) (MEAN±SD)**		**Group-II Condaylar Process Surface Area (mm) (MEAN±SD)**	
	**Right**	**Left**	**Right**	**Left**
**Female **	391.30±2.45	398.40±2.08	454.00±3.46	439.50±4.36*
**Male**	459.00±6.36	457.30±6.49	457.60±5.29	495.40±5.69*

(*p<0.05 significant compared Group-I with Group-II).

**Table 9:** Comparison of mean coronoid process surface area between the genders between the groups.

**Gender**	**Group-I Coronoid Process Surface Area (mm) (MEAN±SD)**		**Group-II Coronoid Process Surface Area (mm) (MEAN±SD)**	
	**Right**	**Left**	**Right**	**Left**
**Female **	495.10±5.19	537.80±6.18*	512.70±1.51	516.20±3.65
**Male**	500.20±8.51	513.50±8.29	492.30±5.34	517.20±4.79*

(*p<0.05 significant compared Group-I with Group-II).
